# Biochemical activity of RAGs is impeded by Dolutegravir, an HIV integrase inhibitor

**DOI:** 10.1038/s41420-020-0281-4

**Published:** 2020-06-12

**Authors:** Namrata M. Nilavar, Amita M. Paranjape, Sathees C. Raghavan

**Affiliations:** Department of Biochemistry, Indian Institute of Science, Bangalore, 560012 India

**Keywords:** HIV infections, HIV infections

## Abstract

HIV is a retrovirus that infects CD4^+^ T lymphocytes in human beings and causes immunodeficiency. In the recent years, various therapies have been developed against HIV, including targeting the HIV specific protein, integrase, responsible for integration of HIV cDNA into host DNA. Although, integrase is specific to HIV, it has functional and structural similarity with RAG1, one of the partner proteins associated with V(D)J recombination, a process by which immune diversity is generated in humans. Currently, there are three HIV integrase inhibitors: Elvitegravir, Dolutegravir, and Raltegravir, in the market which have been approved by the FDA (USA). All three drugs are used in anti-retroviral therapy (ART). Previously, we showed that amongst the HIV inhibitors, Elvitegravir could significantly decrease B cell maturation in vivo and inhibit the physiological activities of RAGs in vitro, unlike Raltegravir. In the present study, we address the effect of second-generation integrase inhibitor, Dolutegravir on RAG activities. Binding and nicking studies showed that, Dolutegravir could decrease the binding efficiency of RAG1 domains and cleavage on DNA substrates, but not as considerably as Elvitegravir. Thus, we show that although the integrase inhibitors such as Elvitegravir show an affinity towards RAG1, the newer molecules may have lesser side-effects.

## Introduction

Acquired immune-deficiency syndrome (AIDS) is the consequence of a period of infection of the human immunodeficiency virus (HIV). The virus specifically targets human CD4^+^ T cells leading to an immunocompromised state, as a result of which the patient succumbs to various infections^1^. Multiple therapies have been developed for combating HIV infection, of which integrase inhibitors are the newest entries. HIV integrase allows insertion of viral DNA into host DNA by catalysing the 3′ processing and strand transfer^2,3^.

**Fig. 1:**
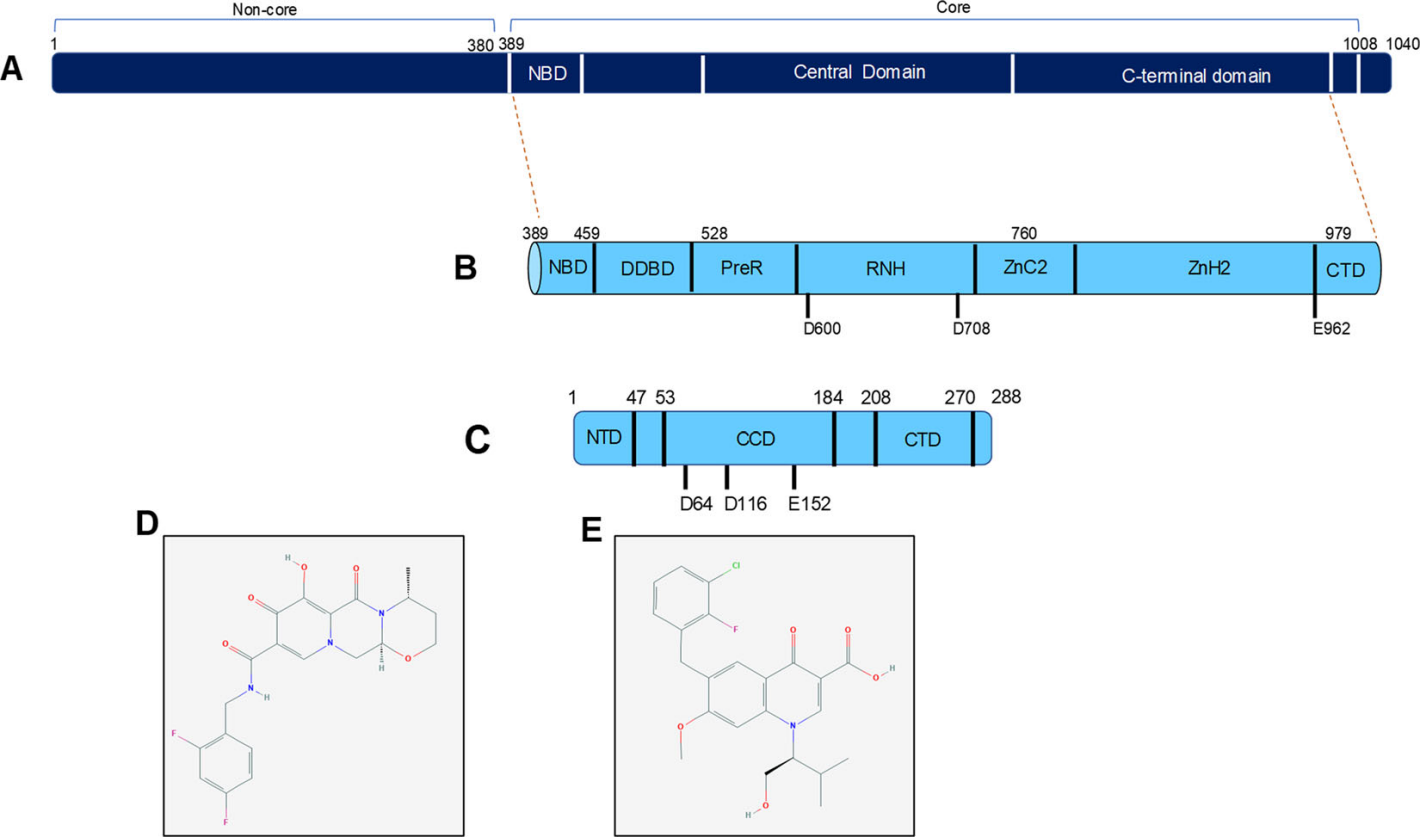
Domain structure of RAG1 and HIV integrase and chemical structure of integrase inhibitors. **a**, **b** Human RAG1 is made of 1040 amino acids. Core RAG1 (amino acids 389–1008) is used for in vitro experiments. Core RAG1 can be divided into three broad domains, namely nonamer binding domain (NBD; amino acids 389–459), central domain (amino acids 528–760) and C-terminal domain (amino acids 760–979). The catalytically active motif of RAG1 lies in the core region, with the D600 and D706 in the central domain and E962 in the C-terminal domain^46,47^. Using cryo-EM microscopy the core region of RAG1 is further characterised into various modules containing NBD (nonamer binding domain), DDBD (DNA binding and dimerisation domain), PreR, RNH, ZnC2, and ZnH2 followed by CTD^46,47^. **c** Human immunodeficiency virus integrase is a small protein consisting of 288 amino acids. It has an N-terminal domain (NTD; amino acids 1–47), catalytic central domain (CCD) (amino acids 53–184) and C-terminal domain (CTD; amino acids 208–270). The catalytically active motif is present in the central domain of HIV integrase and shares similarity with RAG1^48,49^. **d**, **e** Chemical structure of Dolutegravir (**d**)^50^ and Elvitegravir (**e**)^51^ used in the study.

It has been observed that Recombination activating gene 1 (RAG1) and HIV integrase share structural and functional similarities (Fig. 1a–c)^4,5^. RAG1 protein is a part of the RAG complex (RAG1 and RAG2) and is the catalytic partner. The huge amount of antibodies and the variations seen in T-cell receptor (TCR) are a result of V(D)J recombination. Fragments of genomic DNA are cut and joined during the process of V(D)J recombination and RAG complex is the crucial endonuclease responsible for the event. The recombination of DNA assures development of the B and T cells, as well as diversity in both antibodies and TCR. Absence of either RAG1 or RAG2 renders the process incomplete and thus, halts the development of B and T cells. Furthermore, any mutation in RAG1 or its partner protein Recombination activating gene 2 (RAG2) leads to an immunocompromised state resulting in a shorter life-expectancy^6,7^.

Integrase is an essential HIV protein, required for the integration of HIV cDNA into the host genome. It performs 3′ processing of the cDNA following which two reactive 3′ OH are generated^8,9^. These hydroxyl groups carry out a nucleophilic reaction on the host genome resulting in a strand transfer reaction^9^. RAGs, specifically RAG1, carry out the cleavage at the 5′ end of the heptamer of the RSS via transesterification reaction^10,11^. This transesterification reaction is common to both RAGs and the HIV integrase^12^. These two proteins also harbour a common DDE motif which forms the vital catalytic motif utilising Mg^2+^ as a cofactor in the reaction^12^.

Recent years have seen substantial development of HIV integrase inhibitors, specifically inhibitors which target the integrase-viral DNA complex. These inhibitors are termed as integrase strand transfer inhibitors (INSTIs), 5CITEP and p10 were the initial integrase inhibitors to be developed but these showed an inhibition of RAGs physiological properties in vitro^5,13–15^. 5CITEP was discontinued since it was being rapidly cleared from the body by glucuronidation^16^. Raltegravir (commercially known as Isentress®) was the first HIV integrase inhibitor to be approved by the US FDA, which was later followed by the approval of Elvitegravir (Fig. 1e)^17,18^. Both these compounds act by binding to the integrase-viral DNA complex, thus inhibiting the strand transfer reaction but not the 3′-processing reaction^19^. Elvitegravir and Raltegravir, were studied previously and it was shown that Elvitegravir impeded RAG activity in vitro as well as in vivo^20^. Mice fed with Elvitegravir showed a decrease in maturation of B cells^20^. A significant abrogation of RAG1 activity in vitro and in vivo was observed for Elvitegravir, on the other hand Raltegravir caused limited abrogation of RAG1 activity in vitro^20^. There are also reports of high rate of Non-Hodgkin’s lymphoma in patients undergoing treatment with integrase inhibitors as well as early onset of autoimmune diseases^21,22^.

In 2013, another inhibitor named Dolutegravir (DTG) was introduced in the market after US FDA approval, as a second-generation integrase inhibitor (Fig. 1d). It is derived from S/GSK 1265744 and is a nitrogen containing polycyclic compound^23–25^. The compound was recommended at the same dosage as Elvitegravir and has already been formulated in various combinations. Multiple clinical trials (VIKIING 3, VIKING 4 and SPRING 2) have reported that both Raltegravir and Dolutegravir are similar in their safety profiles, but generation of drug resistance is lower for Dolutegravir^26–29^. Also, that the inhibitor Dolutegravir was not inferior compared to the previously two FDA approved inhibitors^27^. Reports suggest that Dolutegravir is an effective anti-retroviral drug for both treatment-naïve and treatment-experienced patients, due to its high virologic suppression, tolerability and once daily regime^25^. Besides, Dolutegravir shows high efficacy toward viral clones resistant to Raltegravir and Elvitegravir^3^. It has been shown that patients can generate cross-resistance to both Raltegravir and Elvitegravir, but not Dolutegravir^30^.

Based on the previous reports of effect of integrase inhibitors on RAG1 functions and in turn V(D)J recombination^5,20^, in the current study, we investigated the effects of Dolutegravir using biochemical assays. Here we report that unlike Elvitegravir, Dolutegravir has several fold lower effect on RAG functions.

## Results

### Dolutegravir inhibits nicking activity of cRAGs, but only at higher concentrations

During the process of V(D)J recombination, RAG1 and RAG2, generally termed as RAG complex, bind to the nonamer of 12 or 23 RSS and cleave at 5′ end of heptamer sequence, resulting in a single-strand break (SSB) or DNA nick. This nick can get converted as a DSB upon hairpin opening by DNA PKcs-Artemis complex. The DSBs are then repaired through Non-homologous end joining.

**Fig. 2:**
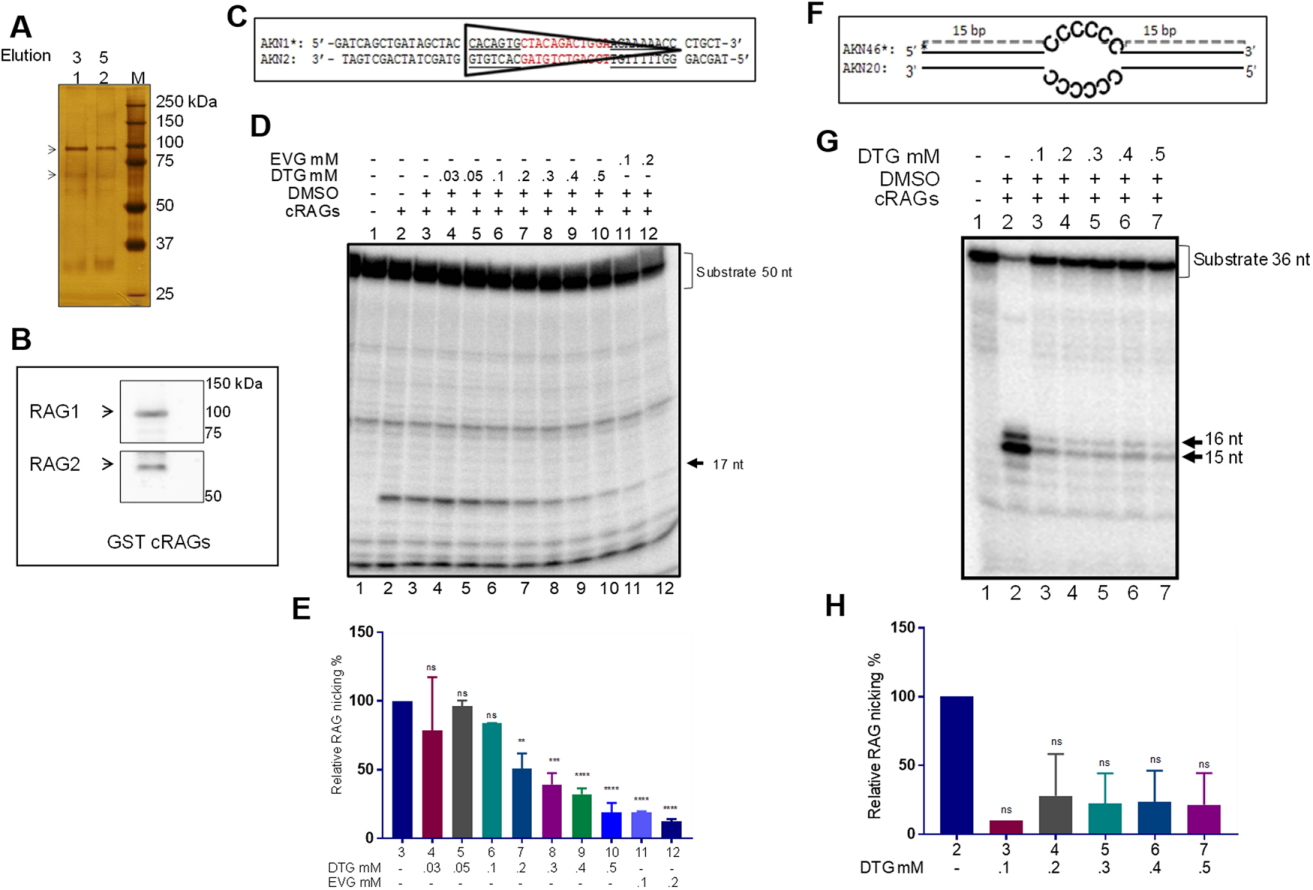
Inhibition of nicking activity of RAGs in presence of Dolutegravir. **a** Silver stained gel showing purified RAG1 and RAG2 from human cells. GST-core RAG1 (~94 kDa) and GST-core RAG2 (~65 kDa) are indicated with arrowheads. **b**. Western blot showing GST-cRAGs. Anti-RAG1 and anti-RAG2 antibodies were used for western blotting. **c** Sequence of DNA substrate containing 12RSS used for the study. **d**, **e** Effect of Dolutegravir on the nicking activity of RAGs on DNA substrate containing 12 RSS. Increasing concentration of Dolutegravir (0.03, 0.05, 0.1, 0.2, 0.3, 0.4 and 0.5 mM) was used for assessing the effect on RAG mediated cleavage on 12 RSS and the products were resolved on a 15% denaturing PAGE. 0.1 and 0.2 mM Elvitegravir was used for comparison of inhibition (**d**). Bar graph representing effect of Dolutegravir on RAG-mediated nicking of 12RSS (*n* = 3). Numbers for each bar corresponds to the lane numbers in the panel **d**. (*P* values ** 0.001, *** 0.0002, **** <0.0001). **f** Sequence and structure of heteroduplex bubble substrate used for the study. **g**. Effect of Dolutegravir on RAG mediated cleavage on heteroduplex DNA. Impact of Dolutegravir on cleavage by cRAG was tested by incubating increasing concentrations of inhibitor (0.1. 0.2, 0.3, 0.4 and 0.5 mM) followed by resolution on a denaturing PAGE. **h** Bar graph representing inhibition of RAG cleavage of heteroduplex DNA by Dolutegravir (*n* = 3). Numbers for each bar corresponds to the lane numbers in panel **g**.

A recent report has shown that Elvitegravir, an integrase inhibitor, can inhibit RAG functions both in vitro and in vivo, but not Raltegravir^20^. In the present study, we have investigated the impact of second-generation integrase inhibitor, Dolutegravir, on V(D)J recombinase and its action. To do this, we overexpressed core RAG1/RAG2 in human kidney epithelial cells (HEK293T), and purified the protein using affinity chromatography. Core RAG1/RAG2 are the essential domains of both the proteins which are shown to catalyse V(D)J recombination both in vitro and inside the cells^31^. Purity was checked using SDS-PAGE followed by silver staining (Fig. 2a) and identity of the proteins were confirmed using western blotting (Fig. 2b). Activity of the proteins were confirmed based on RAG nicking assay, in which a radiolabelled 12 RSS DNA was cleaved by RAGs at the 5′ end of the heptamer leading to the release of 17-nt fragment, which was detected on a denaturing PAGE (not shown).

To investigate the impact of integrase inhibitor on RAGs, radiolabelled double stranded DNA containing 12 RSS (Fig. 2c) was incubated with core-RAG1/RAG2 complex in presence of increasing concentrations (0.03, 0.05, 0.1, 0.2, 0.3, 0.4 and 0.5 mM) of Dolutegravir and products were resolved on a denaturing PAGE (Fig. 2c–e). Results revealed that addition of Dolutegravir lead to a concentration dependent decrease in RAG nicking from 0.2 mM of the inhibitor onwards (Fig. 2d, e). However, there was no effect on RAG induced nicking on RSS in presence of lower concentrations of Dolutegravir (0.03, 0.05 and 0.1; Fig. 2d, e). A decrease in the efficiency of RAG-mediated nicking in presence of higher concentrations of Dolutegravir suggests that the inhibitor interfered with either the binding of RAGs to RSS or the hydrolysis of the phosphodiester bond. On the other hand, Elvitegravir, could significantly inhibit RAG induced nicking at 0.1 mM concentration, which was equivalent to the effect when 0.5 mM of Dolutegravir was used (Fig. 2d, e), indicating that inhibition of RAG functions due to Elvitegravir was much more pronounced than Dolutegravir.

Apart from being a sequence specific nuclease, reports have shown that RAGs can act as structure-specific nuclease, which can cleave non-B forms of DNA leading to chromosomal translocations seen in cancer^32,33^. Previously, we have shown that RAG complex can induce nick at single–double-stranded DNA transition on a heteroduplex DNA^34^. However, the ability of RAGs to bind and cleave at the heteroduplex is not similar to that of 12RSS substrate. Effect of Dolutegravir on RAG mediated cleavage at heteroduplex bubble substrate containing cytosine was evaluated. Results revealed inhibition in RAG mediated nicking on heteroduplex DNA at a concentration of 0.1 mM Dolutegravir onwards (Fig. 2f–h). These results suggest that similar to Elvitegravir, Dolutegravir could also inhibit RAG mediated cleavage on heteroduplex DNA.

### Dolutegravir abrogates binding of RAG1 domains to 12RSS substrate

**Fig. 3:**
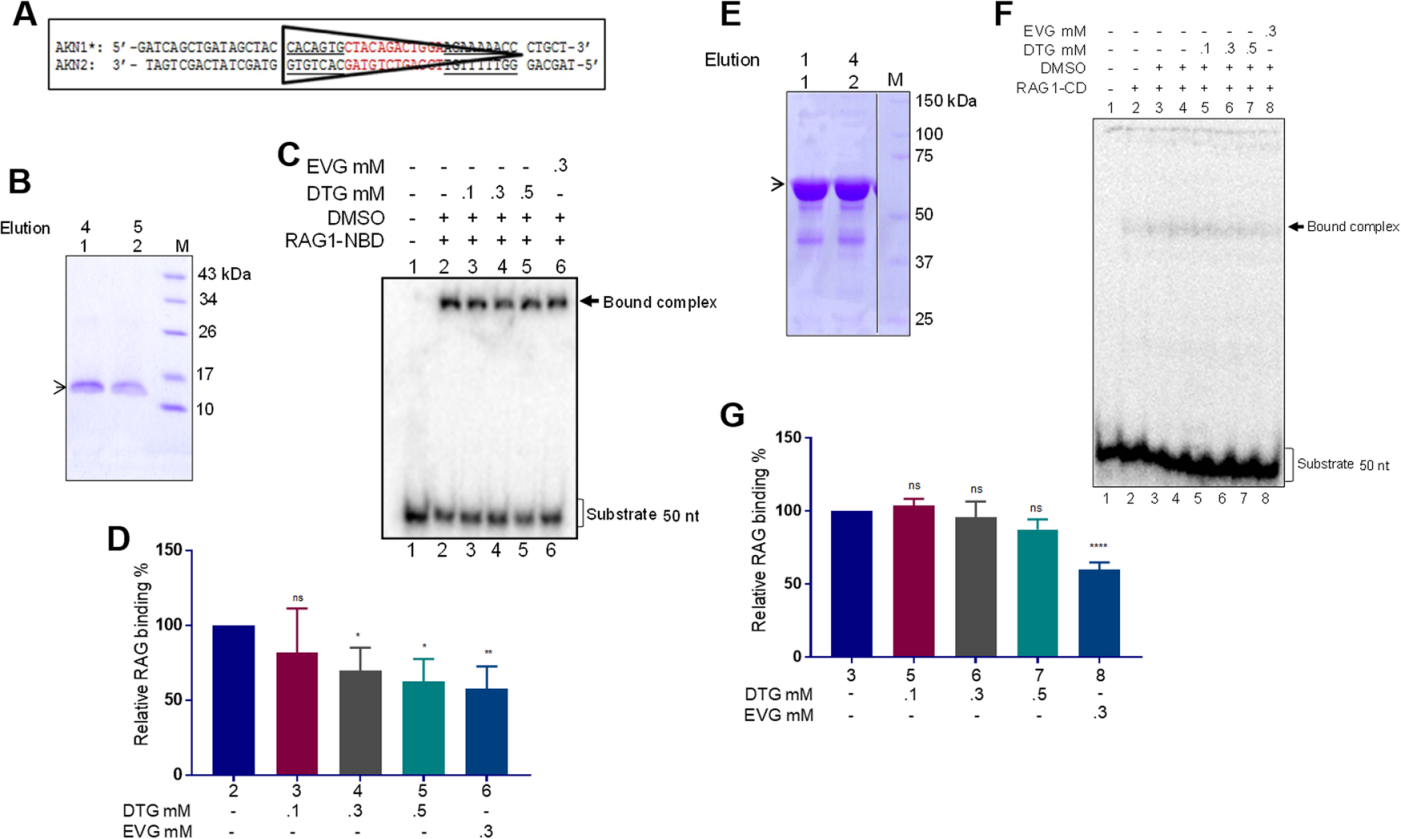
Evaluation of Dolutegravir-mediated inhibition of RAG1 binding on 12RSS. **a** Sequence of 12RSS containing DNA substrate used for the study. **b** SDS-PAGE profile for purified 6X His-tagged RAG1 nonamer binding domain (~11 kDa), marked with an arrowhead. **c**, **d** Effect of Dolutegravir on binding of RAG1-nonamer binding domain to 12RSS substrate was also assessed by incubating increasing concentration of the inhibitor (0.1, 0.3 and 0.5 mM). Products were visualised on a native PAGE and signals were detected (**c**). Bar graph represents quantification based on three independent repeats showing impact of Dolutegravir on NBD binding to RSS (**d**). Numbers for each bar corresponds to the lane numbers in the panel. (*P* values * 0.01 ** 0.001). **e** SDS-PAGE profile for purified RAG1 central domain. The central domain along with MBP tag is ~68 kDa. Protein is seen below 75 kDa marker and is marked with an arrowhead. **f**, **g** Increasing concentrations of Dolutegravir (0.1, 0.3 and 0.5 mM) was incubated with RAG1-central domain, prior to its incubation with 12RSS. Equivalent DMSO concentration was used as vehicle control in the experiment (**f**). Bar graph representing quantification based on three independent repeats for the same is also shown (*n* = 3). Numbers for each bar corresponds to the lane numbers in the panel **g**. (****P values <0.0001).

Nonamer-binding domain of RAG1 is known to bind with RSS^35^. Besides, ZFB present in central domain is also shown to interact with DNA^36^. To investigate if Dolutegravir abrogates binding of various domains of RAG1 to its physiological DNA substrate of recombination signal sequence, we performed binding assays using radiolabelled DNA substrate containing 12RSS (Fig. 3a). Reaction products were resolved on a native polyacrylamide gel to determine shift in the mobility of DNA substrate band due to protein binding and impact of Dolutegravir on DNA–protein interaction.

We performed titration of Dolutegravir along with two domains of RAG1: the nonamer binding domain and central domain. The nonamer binding domain harbours the region of the protein that recognises and binds to the nonamer sequence of the RSS. In contrast, the central domain contains two of the amino acids involved in catalysis. We observed that Dolutegravir exhibited moderate inhibition of binding in a concentration dependent manner when purified NBD of RAG1 was incubated with 12RSS (Fig. 3a–d). However, the efficiency of the inhibition was less than that observed when Elvitegravir was used for the study (Fig. 3c, d). Further the inhibitory effect was much less and restricted to the highest concentration (0.5 mM) when Dolutegravir was tested for its effect on binding of purified RAG1-CD with 12RSS (Fig. 3e–g). Consistent to above observations, the inhibitory effect of Elvitegravir was much higher, than Dolutegravir even in this case (Fig. 3d, g).

### Inhibition of binding at lower concentrations seen using bio-layer interferometry

Results presented above suggest that inhibition of 12RSS nicking by Dolutegravir could be due to the inability of RAG1 NBD to bind to the nonamer sequence when the inhibitor is present. However, the detected level of inhibition in electrophoretic mobility shift assay (EMSA) studies may not explain the extent of inhibition of nicking observed for 12RSS. To investigate the binding efficiency in a quantitative manner, we performed bio-layer interferometry (BLI), a biophysical assay at single molecular level.

**Fig. 4:**
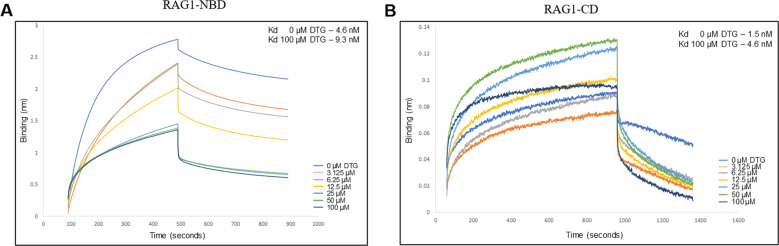
Biolayer-interferometry for measuring bi-molecular interactions. **a**, **b** Biolayer interferometry for RAG1 central domain (RAG1-CD) (**b**) and RAG1 nonamer binding domain (RAG1-NBD) (**a**). Purified CD or NBD was incubated with increasing concentrations of Dolutegravir (3.125, 6.25, 12.5, 25, 50 and 100 µM) and was evaluated using BLI. BLI analysis showed that RAG1-CD and NBD bound with a Kd of 1.5 nM and 4.6 nM, respectively to biotinylated 12 RSS. But in the presence of Dolutegravir a marginal increase in the binding constant was observed; Kd 4.6 nM for RAG1-CD and Kd 9.3 nM for RAG1-NBD.

BLI utilises light refraction to test binding of two molecules. DNA oligomer for 12RSS was added on to a probe using Streptavidin-biotin chemistry. The probe was dipped in solution containing either central or nonamer binding domain of RAG1, with or without Dolutegravir. If Dolutegravir binds to the protein, then there is decrease in binding of the protein to the DNA substrate, which in turn results in a decrease in the interference signal. We incubated, NBD or CD with increasing concentrations of Dolutegravir from 3.125 µM, 6.25 µM, 12.5 µM, 25 µM, 50 µM and 100 µM. The bound 12RSS DNA substrate was then dipped into solution containing protein with or without Dolutegravir. In the presence of Dolutegravir, binding of protein to 12RSS will be hindered and thus, an increase in the Kd of the protein-12RSS binding is expected. Lower Kd values indicate higher affinity of binding. Consistent to EMSA results, we observed elevated binding constant in the presence of Dolutegravir (4.6 nM) for RAG1 central domain (Fig. 4b), compare to RAG1 CD alone (1.5 nM). In contrast to the central domain, for the nonamer binding domain, we saw a two-fold increase in the binding constant. In the absence of Dolutegravir the Kd observed was 4.6 nM, whereas along with 100 µM Dolutegravir, it was 9.3 nM. (Fig. 4a). Since the sensitivity of the technique is higher than EMSA, these results reveal that Dolutegravir can bind to the central domain, which can explain the significant decrease observed in the nicking of 12RSS. These results further confirm that Dolutegravir can interfere with binding of RAG1 to 12RSS, however the observed effect is moderate when compared to Elvitegravir.

## Discussion

HIV integrase and RAG1 have shown to have structural and functional similarity^5,20^ (Fig. 1). It was hypothesised that integrase inhibitors could hinder RAG activity in vivo due to their similarity. In one of the previous studies, it was shown that Elvitegravir indeed inhibited V(D)J recombination^20^. Approximately 70% of mice treated with Elvitegravir showed decrease in maturation of B cells, suggesting inhibition of V(D)J recombination^20^.

In the present study we have examined the second-generation integrase inhibitor, Dolutegravir for its impact on RAG functions, the key enzyme complex associated with V(D)J recombination. Our results suggest that unlike Elvitegravir, impact of Dolutegravir on RAG activity was significantly less. We observed that binding to RAG1 domains by Dolutegravir was significantly less than Elvitegravir. Besides, effect on RAG induced cleavage at RSS was also less for Dolutegravir compared to Elvitegravir. In a previous study another integrase inhibitor, Raltegravir did not show much effect on RAG physiological activity^20^.

Our results reveal that Dolutegravir inhibited RAG binding and nicking only at high concentrations unlike Elvitegravir. EMSA studies in conjunction with Biolayer interferometry results reveal that Dolutegravir could interfere binding of both central domain and nonamer binding domain of RAG1 to 12RSS, thus explaining the observed decrease in RAG induced nicking of 12RSS substrate, but this inhibition was comparatively lower than that seen alongside Elvitegravir.

In summary, the current study along with previous reports^20^, reveal that among the three integrase inhibitors, Elvitegravir shows robust inhibition on RAG functions by inhibiting nicking and binding to its physiological substrate and thus, V(D)J recombination while, Dolutegravir affects the functions only at a moderate level. Importantly, among the three inhibitors, Raltegravir was the least toxic to V(D)J recombination and RAG1 activity^20^. Considering that Dolutegravir is a second-generation inhibitor, the observed impact on RAG functions and thus potentially on the immune system is unexpected. Nevertheless, the observed effects are not too surprising considering the similarity between RAG1 and integrase domains. Regardless, the promising finding is that the nonspecific effect on immune system by Dolutegravir could be several fold lower than Elvitegravir in patients, when one extrapolates our observations. This is indeed propitious and underlines the importance of need for new generation integrase inhibitors. In any case the use of current integrase inhibitors needs to be well-thought-out considering their potential effects on the patient immune repertoire and, appropriate diet and medical interventions need to be used along with these inhibitors.

## Materials and methods

### Enzymes, chemicals, and reagents

Chemicals and reagents used in the study were purchased from Sigma Chemical Co. (USA), and SRL (India). DNA-modifying enzymes were obtained from New England Biolabs (USA). Radioisotope-labelled nucleotides were from BRIT (India). Elvitegravir (GS-9137) was purchased from Selleck Chemicals (Houston, TX, USA). Dolutegravir was purchased from Shanghai Sun-shine Chemical Technology Co. Ltd (China).

### Plasmids

GST tagged human core RAGs expression constructs were gifted by Dr. Michael Lieber, USA. Plasmid construct pRS3, encoding central domain of RAG1 (amino acids 528–760) was kind gift from Dr. Karla Rodgers, USA.

### Cell lines and culture

For expression of core RAG1 and core RAG2, human embryonic kidney cell line expressing Simian virus 40 large T antigen (HEK-293T) was used. DMEM (HiMedia) with high glucose and l-glutamine along with 10% Foetal bovine serum and 1X concentration of penicillin–streptomycin was used for culturing. *E. coli* BL21 (DE3) cells were grown in Luria Bertini broth (HiMedia).

### Oligomeric DNA

Oligomers used for the study were, AKN1, 5′-GATCAGCTGATAG-CTACCACAGTGCTACAGACTGGAACAAAAACCCTGCT-3′; AKN2, 5′-TAGCAGGGTTTTT-GTTCCAGTCTGTAGCACTGTGGTAGCTATCAGCTGAT-3′; AKN 46, 5′-GACCTGAGGG-CGAGCCCCCCCCGAGTAACTTAACAG-3′; and AKN 20, 5′-CTGTTAAGTTACTCGC-CCCCCGCTCGCCCTCAGGTC-3′. AKN1 and AKN2 are complementary oligomers designed to represent 12 RSS, whereas AKN46 and AKN20 are designed to represent heteroduplex C6 bubble following annealing of respective oligomers.

Oligomers were synthesised from Sigma-Aldrich, India and biotinylated oligomers were purchased from Integrated DNA Technologies, USA. Oligomers were purified using denaturing PAGE as described before^25,37^ and dissolved in Tris-EDTA buffer and stored at −20 °C until use.

### 5′ end labelling of oligomers

T4 polynucleotide kinase (NEB) was used to end label oligomers using γ-^32^P ATP in T4 PNK buffer (NEB) as described before^38^. Reaction was incubated for 1 h at 37 °C and then stopped using 10 mM Tris-1 mM EDTA buffer. Labelled substrates were purified using Sephadex G-25 column and then stored at −20 °C until use. Annealing of complementary oligomers was done by boiling oligomers for 10 minutes in the presence of 100 mM NaCl and 1 mM EDTA, in 1:5 ratio (labelled: unlabelled)^37,38^.

### Expression and purification of RAGs

The mammalian expression constructs harbouring core RAG1 (cRAG1, amino acids 384–1040) and core RAG2 (cRAG2, amino acids 1–383) each fused at its N-terminus to the glutathione S-transferase tag was used for expression^39^. The protein was purified as described previously^40,34^ Briefly, HEK293T cells were transfected with RAG constructs at 70% confluency. After 48 h cells were harvested and processed for purification of GST-RAG1 and GST-RAG2 proteins (Fig. 2a, b).

The central and nonamer binding domain of RAG1 were expressed in *Escherichia coli* BL21 (DE3) cells as described earlier^20,34,41^. In short, BL21 (DE3) cells were chemically transformed with specific plasmids for RAG1 central domain (pRS3) and nonamer-binding domain (pDR1). Single colony was amplified and the culture was allowed to grow until OD_600_ reached 0.8 following which IPTG (1 mM) induction was performed for 16 h at 16 °C. The cells were harvested and protein was isolated using affinity chromatography. For RAG1 central domain, amylose column was used as it possessed MBP tag, where as for RAG1 nonamer binding domain, Ni-NTA column was used due to His tag (Fig. 3b, e).

### RAG nicking of oligomeric DNA

RAG nicking buffer (containing 25 mM MOPS (pH 7.0), 30 mM KCl, 30 mM potassium glutamate and 5 mM MgCl_2_) was added to reaction containing labelled oligomeric DNA and purified cRAGs, along with DMSO dissolved Dolutegravir or Elvitegravir^20,34,31^. The reaction was incubated for 1 h at 37 °C and then stopped using formamide dye. Reaction was resolved on a 15% denaturing PAGE containing 7 M urea and 1X TBE buffer. Gels were dried and imaging was done using PhosphorImager FLA9000, Fuji, Japan.

### Electrophoretic mobility shift assay

End labelled annealed oligomers were incubated with RAGs in buffer containing 10 mM Tris-HCl (pH 8.0), 5 mM MgCl_2_, 2 mM DTT, 6% glycerol and 100 mM NaCl^42^. Reactions were incubated for 30 min at room temperature for RAG1-CD and 20 minutes at room temperature for RAG1-NBD along with DMSO dissolved Dolutegravir or Elvitegravir, and then loaded onto 5% native PAGE with 1X TBE buffer^20,40,31^. Gels were dried and imaged using PhosphorImager FLA9000, Fuji, Japan.

### Biolayer interferometry

High Precision Streptavidin Biosensor (SAX) sensors (Forte Bio, USA) were employed for studying the binding of RAG1 domains with or without Dolutegravir (analytes) to biotin tagged proteins (ligand) immobilised onto the (SSA) sensors as described previously^20,43–45^ using the Forte Bio OCTET Red 96 instrument. Before use, all the SAX sensor tips were hydrated in buffer (1X PBS) for 10 min. Biotinylated AKN1 oligomer was used as immobilised ligand to bind to SAX sensors, with the help of Streptavidin-Biotin chemistry. Protein, either nonamer binding domain or central domain of RAG1 was used along with increasing concentration of dolutegravir (3.125, 6.25, 12.5, 25, 50 and 100 µM) as analyte. Programme used: baseline in assay buffer (60 s) followed by association (400 s) of protein with or without inhibitor, followed by dissociation (400 s) in assay buffer, finally regeneration in 2 M NaCl. EMSA buffer was used as assay buffer in the experiment. Reference sensor without immobilised ligand was subjected to the same procedure as the sample sensors, and then used for subtraction of the background signals during analysis. Dissociation constant was calculated using 1:1 global fit analysis.

### Statistical analysis

Statistical analysis was done using one-way ANOVA or by using Student’s *t* test by comparing multiple conditions with control sample set using GraphPad Prism.
